# Urethral Interfractional Geometric and Dosimetric Variations of Prostate Cancer Patients: A Study Using an Onboard MRI

**DOI:** 10.3389/fonc.2022.916254

**Published:** 2022-07-15

**Authors:** Jonathan Pham, Ricky R. Savjani, Stephanie M. Yoon, Tiffany Yang, Yu Gao, Minsong Cao, Peng Hu, Ke Sheng, Daniel A. Low, Michael Steinberg, Amar U. Kishan, Yingli Yang

**Affiliations:** ^1^ Department of Radiation Oncology, University of California, Los Angeles, Los Angeles, CA, United States; ^2^ Department of Radiology, University of California, Los Angeles, Los Angeles, CA, United States; ^3^ Department of Urology, University of California, Los Angeles, Los Angeles, CA, United States

**Keywords:** MR-guided radiation therapy (MRgRT), prostate cancer, dosimetry, toxicity, urethra

## Abstract

**Purpose:**

For a cohort of prostate cancer patients treated on an MR-guided radiotherapy (MRgRT) system, we retrospectively analyzed urethral interfractional geometric and dosimetric variations based on onboard MRIs acquired at different timepoints and evaluated onboard prostatic urethra visualization for urethra-focused online adaptive RT.

**Methods:**

Twenty-six prostate cancer patients were prospectively scanned on a 0.35-T MRgRT system using an optimized T2-weighted HASTE sequence at simulation and final fraction. Two radiation oncologists (RO1 and RO2) contoured the urethras on all HASTE images. The simulation and final fraction HASTE images were rigidly registered, and urethral interobserver and interfractional geometric variation was evaluated using the 95th percentile Hausdorff distance (HD95), mean distance to agreement (MDA), center-of-mass shift (COMS), and DICE coefficient. For dosimetric analysis, simulation and final fraction HASTE images were registered to the 3D bSSFP planning MRI and 3D bSSFP final setup MRI, respectively. Both ROs’ urethra contours were transferred from HASTE images for initial treatment plan optimization and final fraction dose estimation separately. Stereotactic body radiotherapy (SBRT) plans, 40 Gy in 5 fractions, were optimized to meet clinical constraints, including urethral V42Gy ≤0.03 cc, on the planning MRI. The initial plan was then forward calculated on the final setup MRI to estimate urethral dose on the final fraction and evaluate urethral dosimetric impact due to anatomy change.

**Results:**

The average interobserver HD95, MDA, COMS, and DICE were 2.85 ± 1.34 mm, 1.02 ± 0.36 mm, 3.16 ± 1.61 mm, and 0.58 ± 0.15, respectively. The average interfractional HD95, MDA, COMS, and DICE were 3.26 ± 1.54 mm, 1.29 ± 0.54 mm, 3.34 ± 2.01 mm, and 0.49 ± 0.18, respectively. All patient simulation MRgRT plans met all clinical constraints. For RO1 and RO2, 23/26 (88%) and 21/26 (81%) patients’ final fraction estimated urethral dose did not meet the planned constraint. The average urethral V42Gy change was 0.48 ± 0.58 cc.

**Conclusion:**

Urethral interfractional motion and anatomic change can result in daily treatment violating urethral constraints. Onboard MRI with good visualization of the prostatic urethra can be a valuable tool to help better protect the urethra through patient setup or online adaptive RT.

## Introduction

Stereotactic body radiotherapy (SBRT) is now a widely accepted standard-of-care option for localized prostate cancer (1). Despite an overall highly favorable safety profile, SBRT late (13.3%) grade ≥2 genitourinary (GU) toxicity rates remain a significant challenge (2–5). Past efforts for reducing late GU toxicities have been focused on bladder sparing (6). However, urethral injury is also a significant contributor to GU toxicity (7, 8). The urethra can be constrained below the prescription dose (urethra sparing) or above (hotspot limitation). Prospective SBRT trials have reported allowable urethra doses ranging between 34.7 and 52.5 Gy in 5 fractions (9, 10). Leeman et al. analyzed patients enrolled in trials for SBRT and showed that an increase in the maximum urethral dose metric (MUDM) correlated to an increase in acute (≤3 months) and late (>3 months) grade ≥2 GU toxicity rates (8). While urethral sparing approaches are appealing from the standpoint of limiting toxicity, postradiation patterns of failure studies have suggested that periurethral recurrences are common, and therefore, hotspot limitation may be a better goal for minimizing toxicity while maintaining efficacy (11).

In addition to urethral dose constraints, urethra delineation uncertainty and intrafractional/interfractional motion can also contribute to GU toxicity. Delineating the urethra on computed tomography (CT) images is non-trivial due to the lack of contrast between the urethra and prostate (12). Foley catheters have been used to delineate the urethra on planning CTs; however, the catheter can also displace and deform the urethra, resulting in urethra misposition (13, 14). Alternatively, magnetic resonance images (MRIs) can be acquired and registered to planning CTs for urethra delineation (15). Diagnostic 3T T2-weighted MRI has shown good urethra visualization and low interobserver urethra contouring variation (16, 17). However, contouring uncertainty from cross-modality registration adds uncertainties (18). Moreover, the shape and location of the urethra may change between diagnostic MRI and planning CT acquisitions, which are often acquired on different days with different patient positions. As for urethra intrafractional/interfractional motion, little has been studied and its impact on urethral dose is unknown due to limited urethra visualization tools.

Recently, advancements in MR-guided radiation therapy (MRgRT) and the development of MR linear accelerators (MR-LINAC), equipped with onboard MRI, have allowed the application of MRI for prostate treatment planning, adaptation, and monitoring. MRI provides high soft-tissue contrast for accurate tumor and critical structure delineation (19). MR-Linac’s onboard MRIs allow for fiducial-free daily patient setup and interfractional MR-guided online adaptive radiation therapy (MRgART), where initial treatment plans can be recalculated or reoptimized based on the patient’s daily anatomy (20). Real-time cine MR can also be acquired during treatment delivery to monitor intrafraction motion and gate treatment (21). Consistent and frequent radiation-free MR imaging, throughout patient treatment, enables the use of smaller planning margins and improved critical structure sparing (5). Furthermore, the MRgRT workflow minimizes cross-modality and cross-system registration errors as the MRIs are acquired on the same system with the patient in the treatment position.

Currently, it is standard practice to acquire a 3D balanced steady-state free precession (bSSFP) MRI for MRgRT treatment planning and daily patient setup using the ViewRay MRIdian MR-Linac (ViewRay Inc., Oakwood Village, OH, USA). Clinical bSSFP is intrinsically fast and has a high signal-to-noise ratio (SNR). However, it is T2/T1-weighted and provides lower urethral contrast than T2-weighted scans (22). As a result, at our institution, a T2-weighted MRI sequence is optimized and performed at the end of patient MR simulation for urethra delineation (22). Due to time constraints, T2-weighted MRIs are not acquired for daily patient setup and are acquired with a smaller FOV covering only the prostate gland. Herein, we analyze interobserver variability as well as geometric and dosimetric changes in the urethra between the simulation scan and the final fraction of SBRT in a cohort of prospectively treated patients to determine the clinical significance of onboard urethra visualization for urethra-focused MRgART.

## Methods and Materials

This study was conducted according to the guidelines of the Declaration of Helsinki and approved by the Institutional Review Board of the University of California, Los Angeles, IRB #17-001064, on December 6, 2017. Twenty-six prostate cancer patients undergoing MRgRT SBRT between June 2020 and June 2021 were prospectively included. Prior to patient simulation and each treatment fraction, patients were instructed to follow the institutional bladder filling and rectum emptying protocol. For CT simulation, patients were immobilized with a vacuum bag and a pelvic CT was acquired on a 16-slice CT scanner (Sensation Open, Siemens Medical Solutions, Erlangen, Germany). For MR simulation and before each treatment fraction, a clinical bSSFP MRI was acquired on a 0.35-T MR-Linac system (ViewRay MRIdian, ViewRay Inc., Cleveland, OH, USA) using the same immobilization device. Additionally, a urethra-specific T2-weighted 3D half-Fourier acquisition single-shot turbo spin-echo (3D HASTE) was acquired at simulation (HASTE 1) and at the end of the final treatment fraction (HASTE 2). Urethra imaging was only acquired at two timepoints due to clinical time constraint. 3D HASTE sequence parameters are as follows: repetition time (TR) = 1,800 ms, echo time (TE) = 246 ms, voxel size 1.5 mm isotropic, FOV = 227 × 400 mm^2^, number of slices = 40, number of averages = 6, and acquisition time = 8:06 min. A more detailed explanation of 3D HASTE sequence optimization can be found in Pham et al. (22).

The simulation clinical bSSFP MRI serves as the primary treatment planning image (planning MRI). An attending physician contoured the prostate gland as the clinical target volume (CTV) and all critical structures on the planning MRI in MIM Software (Cleveland, OH, USA). Due to high MRI prostate visualization and MRgRT daily/real-time image guidance, the planning target volume (PTV) was constructed by isotropically expanding the CTV by 2 mm. Two radiation oncologists (RO1 and RO2) independently contoured the prostatic urethras on both HASTE 1 and HASTE 2 for all patients. Prostatic urethra contours were cropped to be within the PTV. HASTE 1 and 2 were rigidly registered in MIM Software using box-based assisted alignment on the prostate. Afterward, a medical physicist checked the registration and manual translational/rotational adjustments were made if necessary. Urethral interobserver and interfractional geometric variation was evaluated using the 95th percentile Hausdorff distance (HD95), mean distance agreement (MDA), center-of-mass shift (COMS), and DICE coefficient. A DICE coefficient score of >0.70 reflects a good spatial and volumetric agreement between observers or no geometrical change between imaging fractions (17). Additionally, HASTE 1 and 2 bladder volumes were estimated, and the association between bladder volume change and urethral motion was assessed using regression analysis. Due to HASTE images’ limited FOV, complete bladder volume could not be measured, and as a result, a surrogate area index (Area = A × B) was used, in which the long axis (A) and the perpendicular short axis (B) of the bladder in the central sagittal plane were measured.

Furthermore, each RO qualitatively scored the urethra visibility of each image on a 4-point scale: 1 = no conspicuity; 2 = some conspicuity, the urethra can be identified, but not very clear; 3 = good conspicuity, the urethra can be identified clearly; and 4 = excellent conspicuity. RO1 and RO2’s urethra visibility scores were compared using the Wilcoxon signed-rank test with a significance level of 0.05.

For dosimetric analysis, HASTE 1 and HASTE 2 were rigidly registered to their respective clinical bSSFP MRI. Both RO’s urethra contours were transferred separately from HASTE images for treatment planning and dose estimation. For each RO, an MRgRT treatment plan was generated on the planning MRI using clinical contours and their respective HASTE 1 urethra contours. MRgRT plans were prescribed to deliver 40 Gy to 95% of PTV [5 fractions (Fx); 8 Gy/Fx]. Each plan was optimized to meet clinical constraints (**Table 1**), including a urethral hotspot limiting constraint (V42Gy ≤ 0.03 cc). Urethral hotspot limitation constraint was prioritized over urethral sparing to maintain treatment efficacy and reduce the risk of disease recurrence. The dose was calculated on the planning MRI with deformably registered electron density information from simulation CT using the MRgRT treatment planning system. Afterward, the final fraction urethral dose was estimated by performing a forward calculation of the initial plan onto the final fraction patient setup bSSFP MRI. Urethral constraint, mean dose, D0.03cc, V42Gy, and PTV mean dose change between simulation and final fraction were evaluated. Simulation and final fraction dose parameters were compared using paired t-test with a significance level of 0.05.

**Table 1:** Clinical constraints for prostate patients.

Constraint	
PTV V40Gy	≥95%
PTV V42Gy	<30%
Rectum V20Gy	<50%
Rectum V36Gy	<10%
Rectum V40Gy	<5%
Bowel V20Gy	<30 cc
Urethra V42Gy	≤0.03 cc
Bladder V20Gy	<40%
Bladder V39Gy	<4 cc
Bladder V40Gy	<10%

## Results

The average time between simulation and final fraction imaging was 21.4 ± 4.6 days. RO1’s and RO2’s average qualitative urethra visibility scores were 1.8 ± 0.7 and 3.2 ± 0.7, respectively. RO2 scored urethra visibility significantly greater than RO1 (*p* < 0.05). The average HD95, MDA, COMS, and DICE between RO1 and RO2’s urethra contours were 2.85 ± 1.34 mm, 1.02 ± 0.36 mm, 3.16 ± 1.61 mm, and 0.58 ± 0.15.

**Figure 1:**
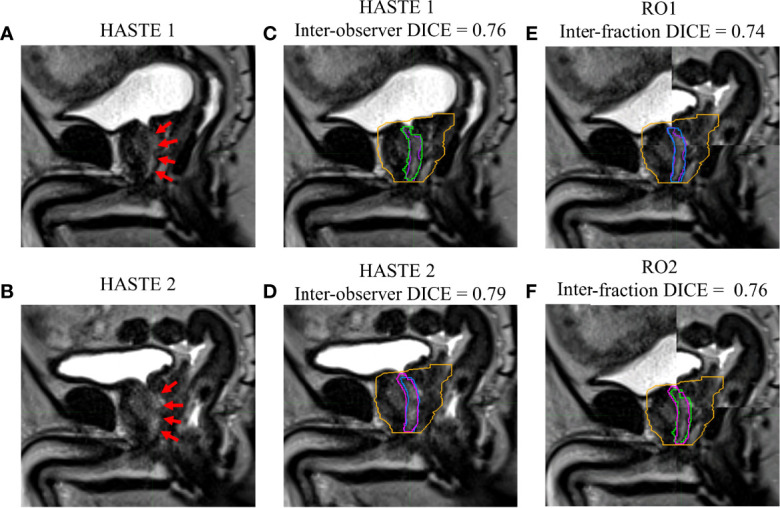
Patient A’s **(A)** simulation (HASTE 1) and **(B)** final fraction (HASTE 2) urethra images (red arrows pointing to the urethra). **(C)** Interobserver urethra contour agreement between RO1 (purple) and RO2 (green) for HASTE 1. **(D)** Interobserver urethra contour agreement between RO1 (blue) and RO2 (pink) for HASTE 2. Planning target volume (PTV) is contoured in orange. Interfractional urethra changes for **(E)** RO1 and **(F)** RO2 on fused (checkerboard layout) HASTE 1 and 2 images.

**Figure 2:**
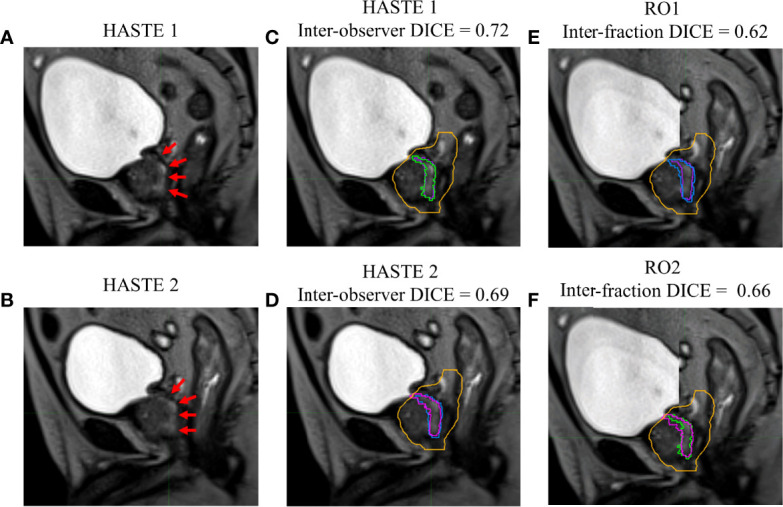
Patient B’s **(A)** simulation (HASTE 1) and **(B)** final fraction (HASTE 2) urethra images (red arrows pointing to the urethra). **(C)** Interobserver urethra contour agreement between RO1 (purple) and RO2 (green) for HASTE 1. **(D)** Interobserver urethra contour agreement between RO1 (blue) and RO2 (pink) for HASTE 2. Planning target volume (PTV) is contoured in orange. Interfractional urethra changes for **(E)** RO1 and **(F)** RO2 on fused (checkerboard layout) HASTE 1 and 2 images.

**Figure 3:**
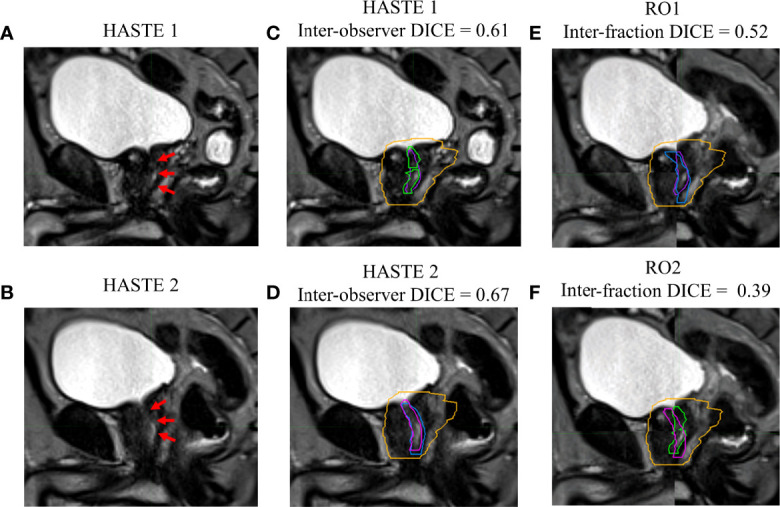
Patient C’s **(A)** simulation (HASTE 1) and **(B)** final fraction (HASTE 2) urethra images (red arrows pointing to the urethra). **(C)** Interobserver urethra contour agreement between RO1 (purple) and RO2 (green) for HASTE 1. **(D)** Interobserver urethra contour agreement between RO1 (blue) and RO2 (pink) for HASTE 2. Planning target volume (PTV) is contoured in orange. Interfractional urethra changes for **(E)** RO1 and **(F)** RO2 on fused (checkerboard layout) HASTE 1 and 2 images.

**Figure 4:**
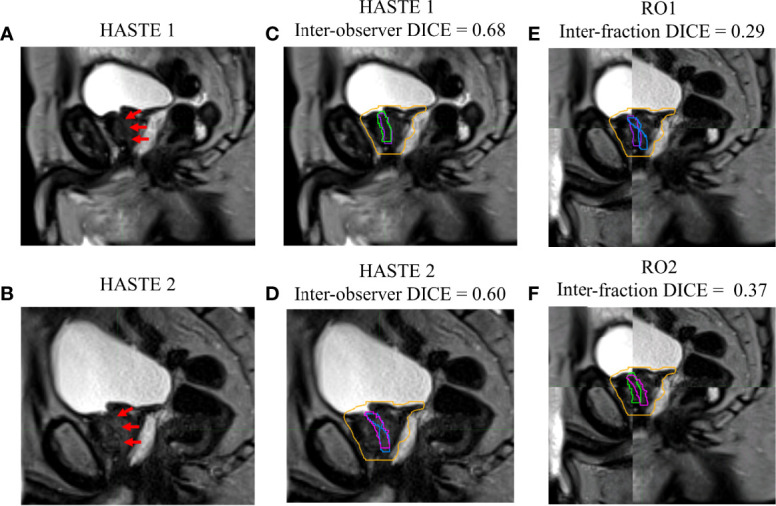
Patient D’s **(A)** simulation (HASTE 1) and **(B)** final fraction (HASTE 2) urethra images (red arrows pointing to the urethra). **(C)** Interobserver urethra contour agreement between RO1 (purple) and RO2 (green) for HASTE 1. **(D)** Interobserver urethra contour agreement between RO1 (blue) and RO2 (pink) for HASTE 2. Planning target volume (PTV) is contoured in orange. Interfractional urethra changes for **(E)** RO1 and **(F)** RO2 on fused (checkerboard layout) HASTE 1 and 2 images.

**Figures 1**–**4** show four prostate patients’ (*Patients* A–D) HASTE 1 and 2 with RO1 and RO2 contours. Patients A–D showed ok–good interobserver contour agreement (DICE > 0.60). Patients A and B showed minimal urethral interfractional change (DICE > 0.62), while patients C and D showed significant urethral interfractional change (DICE < 0.54). The combined RO average HD95, MDA, COMS, and DICE between simulation and final fraction urethra contours for all patients were 3.26 ± 1.54 mm, 1.29 ± 0.54 mm, 3.34 ± 2.01 mm, and 0.49 ± 0.18, respectively. No correlation between urethral motion and the bladder volume surrogate was observed (*R* ^2^ < 0.1).

All patient simulation MRgRT plans met all clinical constraints, including urethral hotspot constraints. The combined RO average simulation urethral mean dose, D0.03cc, V42Gy, and PTV mean dose were 40.69 ± 0.37 Gy, 41.83 ± 0.21 Gy, 0.02 ± 0.01 cc, and 41.29 ± 0.22 Gy, respectively. However, for RO1 and RO2, 23/26 (88%) and 21/26 (81%) patients’ final fraction estimated urethral dose did not meet V42Gy ≤0.03 cc. The combined RO average final fraction urethral mean dose, D0.03cc, V42Gy, and PTV mean dose were 41.10 ± 0.68 Gy, 42.62 ± 0.72 Gy, 0.50 ± 0.58 cc, and 40.84 ± 0.65 Gy, respectively. The final fraction urethral dose parameters were significantly greater than the simulation (*p* < 0.05), whereas the PTV dose parameters were significantly less (*p* < 0.05). The combined RO average urethral mean dose, D0.03cc, V42Gy, and PTV mean dose change were 0.41 ± 0.60 Gy, 0.79 ± 0.74 Gy, 0.48 ± 0.58 cc, and −0.45 ± 0.71, respectively. Overall, dose parameters and urethral constraint change were consistent for both ROs.

**Figure 5:**
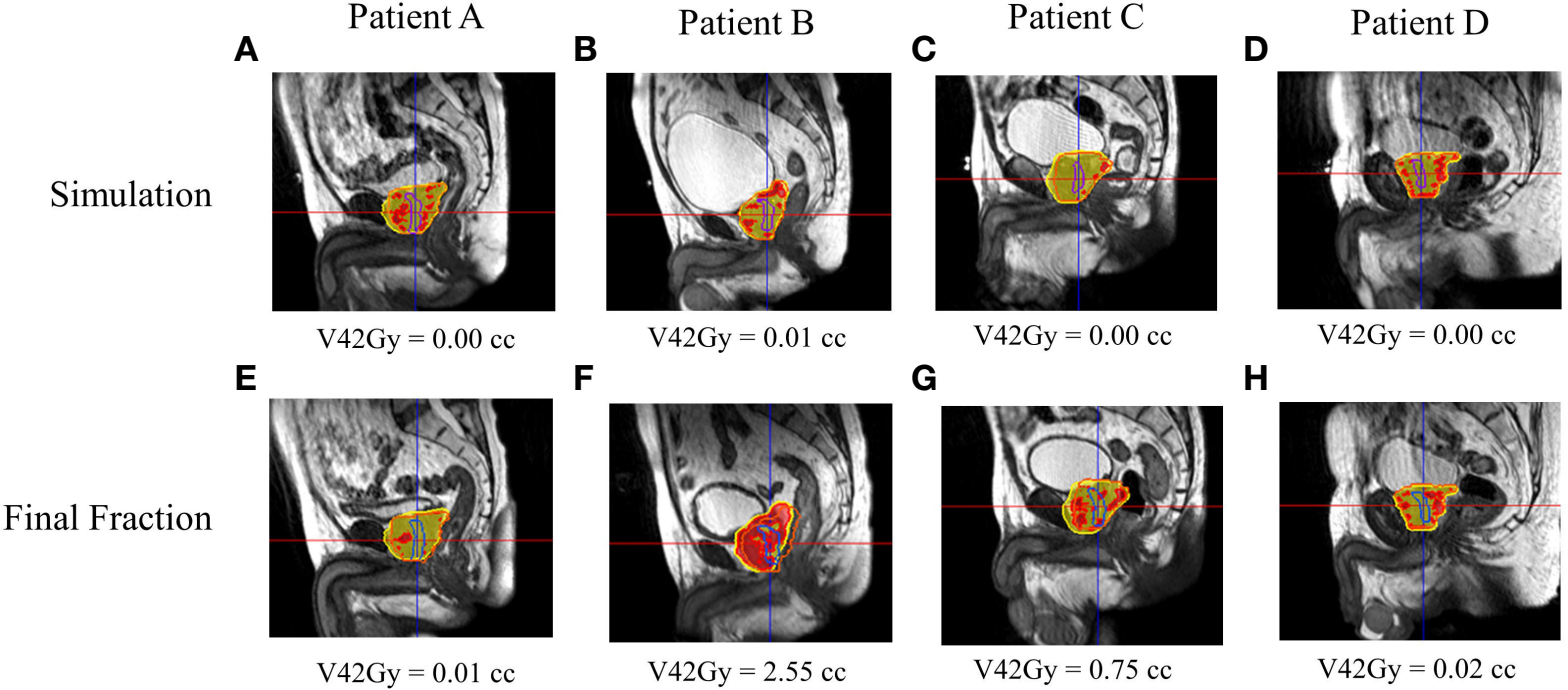
RO1’s calculated and estimated dose and urethral V42Gy for patient A–D’s simulation **(A–D)** and final fraction **(E–H)** bSSFP. RO1 simulation/final HASTE urethra contour—purple/blue. Orange contour—PTV. Red—105% (42 Gy) isodose region, yellow—95% (38 Gy) isodose region.

**Figure 6:**
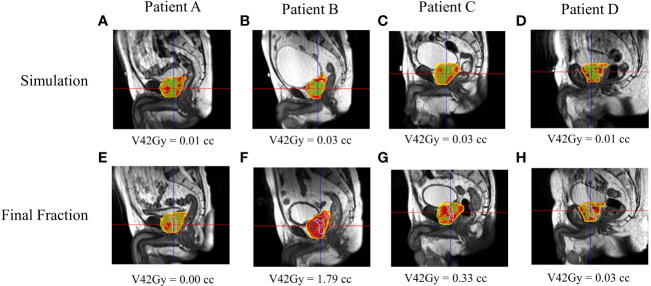
RO2’s calculated and estimated dose and urethral V42Gy for patient A–D’s simulation **(A–D)** and final fraction **(E–H)** bSSFP. RO2 simulation/final HASTE urethra contour—purple/blue. Orange contour—PTV. Red—105% (42 Gy) isodose region, yellow—95% (38 Gy) isodose region.

**Figures 5**, **6** show both ROs’ patients’ A–D calculated (simulation) and estimated (final fraction) dose and urethra V42Gy. Patient A demonstrated minimal geometric urethral change and, as a result, little urethral dose change. Alternatively, Patient B showed minimal geometric urethral change but significant urethral dose changes due to other anatomical changes such as differential bladder filling. Patient C exhibited significant geometric urethral change, resulting in the urethra moving into hotspot regions. Patient D showed significant geometric urethral change but little dose change, demonstrating the importance of hotspot location and robustness of each MRgRT IMRT plan.

## Discussion

This study evaluated prostate cancer patients’ interfractional urethral geometric and dosimetric changes. Significant geometric and spatial urethral changes between simulation and the final fraction were noticed, indicating the potential need for daily urethral imaging to achieve better urethra protection by limiting urethral hotspots in MRgRT treatment planning and delivery. Our study reveals that the efficacy of urethral hotspot-limiting constraints depends on interfractional urethral geometric and anatomic changes as more than 80% of patients had a failing final fraction urethra V42Gy constraint. In other words, interfractional urethral geometric changes can result in a significant volume of the urethra moving into planned hotspot regions as shown in **Figures 5**, **6**. Additionally, interfractional anatomical changes such as the bladder filling variation and prostate swelling can significantly alter the planned dose distribution and result in a higher urethral dose (23). Currently, there is no well-established dosimetric constraint for the urethra. Prostate cancer patients were prescribed a 5Fx × 8-Gy SBRT schedule to the PTV, which is a higher dose than the more common, lower dose 5Fx × 7.25Gy schedule. In principle, a lower prescription dose may have a lower likelihood of GU toxicity; however, urethral hotspots remain a concern for the 5Fx × 7.25Gy schedule as the CTV, containing the urethra, is still prescribed to receive 40 Gy (3). The MRgART workflow with onboard urethral imaging may be valuable to account for the daily urethral change as shown in this study, and if necessary, treatment reoptimization may be utilized to replan and reduce daily urethral hotspots and, as a result, GU toxicity.

This study had several limitations. First, there is a lack of urethra ground truth to reference, and as a result, interfractional urethral geometric and dosimetric changes are reported as relative changes. Currently, there is no gold-standard ground truth for urethra localization at the time of treatment. Nonetheless, the much improved soft-tissue contrast with the urethral-specific MRI makes us more confident in urethra localization. Second, urethra qualitative visibility with our current MRI sequence varied considerably between patients and between observers. Interpatient urethral visibility variance may be due to varying amounts of residual urine in the prostatic urethra, surrounding fat and motion/ghosting artifacts, as well as nearby prostatic hyperplasia compressing the prostatic urethra (16). Interobserver urethral visibility variance can also be due to different observer experiences. Further MRI sequence and imaging protocol optimization is necessary to achieve more robust urethral visualization. Third, the reported urethral MRI sequence took 7–8 min, which may be impractical for the already time-intensive MRgART workflow. The long urethral scan time can increase the chance of unwanted patient motion and anatomical changes. Therefore, future work will explore MR sequence acceleration strategies. Lastly, due to long urethral imaging times and clinical time constraints, urethra images were only acquired at simulation and at the final fraction, which limits the accuracy of the reported urethral interfractional geometric and dosimetric changes or variations. Despite this, a total of 26 prostate cancer patients were recruited, and the reported results of the entire cohort can be used to estimate urethral interfractional variations.

## Conclusion

Interfractional urethral geometric or anatomical changes can result in clinically significant urethral dose change for prostate cancer patients treated with urethral hotspot-limiting MRgRT plans, potentially contributing to an increased urethral dose. The MRgART workflow with onboard urethral imaging may be used to reduce daily urethral hotspots and, as a result, GU toxicity.

## Data Availability Statement

The datasets presented in this article are not readily available because the dataset will not be made available due to patient privacy concerns. Requests to access the datasets should be directed to jonathanpham@mednet.ucla.edu.

## Ethics Statement

The studies involving human participants were reviewed and approved by Institutional Review Board of University of California, Los Angeles, IRB #17-001064. The patients/participants provided their written informed consent to participate in this study.

## Author Contributions

JP was the lead author and contributed in data collection, data analysis, manuscript drafting, table/figure creation, and manuscript revision. RS and SY contributed in data collection and manuscript review. TY contributed in data analysis and manuscript review. YG, MC, PH, KS, DL, MS, and AK aided in manuscript review. YY was the senior author who developed the concept of the study and revised the manuscript. All authors contributed to the article and approved the submitted version.

## Conflict of Interest

YY and MC have received honoraria and consulting fees from ViewRay. AK has received honoraria, consulting fees, and research funding from ViewRay, Inc., as well as honorarium and consulting fees from Varian Medical Systems, Inc.; he also holds low-value stock in ViewRay, Inc. MS has received a consulting fee from ViewRay. PH has received consulting fees and research funding from ViewRay. DL has received consulting fees from ViewRay and a grant from Varian.

ViewRay, Inc. and Varian Medical Systems, Inc. were not involved in the study design, collection, analysis, interpretation of data, the writing of this article or the decision to submit it for publication.

The remaining authors declare that the research was conducted in the absence of any commercial or financial relationships that could be construed as a potential conflict of interest

## Publisher’s Note

All claims expressed in this article are solely those of the authors and do not necessarily represent those of their affiliated organizations, or those of the publisher, the editors and the reviewers. Any product that may be evaluated in this article, or claim that may be made by its manufacturer, is not guaranteed or endorsed by the publisher.

## References

[1] Mohler JL Antonarakis ES Armstrong AJ D’Amico AV Davis BJ Dorff T. Prostate Cancer, Version 2.2019, NCCN Clinical Practice Guidelines in Oncology. J Natl Compr Canc Netw (2019) 17:479–505. doi: 10.6004/JNCCN.2019.0023 31085757

[2] Kishan AU Dang A Katz AJ Mantz CA Collins SP Aghdam N. Long-Term Outcomes of Stereotactic Body Radiotherapy for Low-Risk and Intermediate-Risk Prostate Cancer. JAMA Netw Open (2019) 2:e188006–e188006. doi: 10.1001/JAMANETWORKOPEN.2018.8006 30735235 PMC6484596

[3] Brand DH Tree AC Ostler P van der Voet H Loblaw A Chu W. Intensity-Modulated Fractionated Radiotherapy Versus Stereotactic Body Radiotherapy for Prostate Cancer (PACE-B): Acute Toxicity Findings From an International, Randomised, Open-Label, Phase 3, Non-Inferiority Trial. Lancet Oncol (2019) 20:1531–43. doi: 10.1016/S1470-2045(19)30569-8/ATTACHMENT/F9776432-045B-4658-9ED8-001722D244A2/MMC1.PDF PMC6838670 31540791

[4] Widmark A Gunnlaugsson A Beckman L Thellenberg-Karlsson C Hoyer M Lagerlund M. Ultra-Hypofractionated Versus Conventionally Fractionated Radiotherapy for Prostate Cancer: 5-Year Outcomes of the HYPO-RT-PC Randomised, Non-Inferiority, Phase 3 Trial. Lancet (2019) 394:385–95. doi: 10.1016/S0140-6736(19)31131-6/ATTACHMENT/BE42A27F-79B9-4D7D-93AE-F4FF9FDFE327/MMC1.PDF 31227373

[5] Ma TM Lamb JM Casado M Wang X Basehart TV Yang Y. Magnetic Resonance Imaging-Guided Stereotactic Body Radiotherapy for Prostate Cancer (Mirage): A Phase Iii Randomized Trial. BMC Cancer (2021) 21. doi: 10.1186/S12885-021-08281-X PMC8114498 33975579

[6] Cheung MR Tucker SL Dong L de Crevoisier R Lee AK Frank S. Investigation of Bladder Dose and Volume Factors Influencing Late Urinary Toxicity After External Beam Radiotherapy for Prostate Cancer. Int J Radiat Oncol Biol Phys (2007) 67:1059–65. doi: 10.1016/J.IJROBP.2006.10.042 PMC2081969 17241755

[7] Wang K Mavroidis P Royce TJ Falchook AD Collins SP Sapareto S. Prostate Stereotactic Body Radiation Therapy: An Overview of Toxicity and Dose Response. Int J Radiat Oncol Biol Phys (2021) 110:237–48. doi: 10.1016/J.IJROBP.2020.09.054/ATTACHMENT/3115C618-1565-42D0-9072-4BE340E8138D/MMC1.DOCX PMC8053668 33358229

[8] Leeman JE Chen YH Catalano P Bredfeldt J King M Mouw KW. Radiation Dose to the Intraprostatic Urethra Correlates Strongly With Urinary Toxicity After Prostate Stereotactic Body Radiation Therapy: A Combined Analysis of 23 Prospective Clinical Trials. Int J Radiat Oncol Biol Phys (2022) 112:75–82. doi: 10.1016/J.IJROBP.2021.06.037 34711459

[9] Zilli T Jorcano S Bral S Rubio C Bruynzeel AME Oliveira A. Once-A-Week or Every-Other-Day Urethra-Sparing Prostate Cancer Stereotactic Body Radiotherapy, a Randomized Phase II Trial: 18 Months Follow-Up Results. Cancer Med (2020) 9:3097–106. doi: 10.1002/CAM4.2966 PMC7196054 32160416

[10] Hannan R Tumati V Xie X-J Cho LC Kavanagh BD Brindle J. Stereotactic Body Radiation Therapy for Low and Intermediate Risk Prostate Cancerdresults From a Multi-Institutional Clinical Trial *. Eur J Cancer (2016) 59:142–51. doi: 10.1016/j.ejca.2016.02.014 27035363

[11] Takeda T Tin AL Corradi RB Mamoor M Benfante NE Sjoberg DD. Topography of Prostate Cancer Recurrence After Radiation Therapy: A Detailed Mapping Study of Salvage Radical Prostatectomy Specimens. Eur Urol (2018) 73:488–90. doi: 10.1016/J.EURURO.2017.08.005 PMC6050029 28851581

[12] Waterman FM Dicker AP. Determination of the Urethral Dose in Prostate Brachytherapy When the Urethra Cannot Be Visualized in the Postimplant CT Scan. Med Phys (2000) 27:448–51. doi: 10.1118/1.598912 10757596

[13] Litzenberg DW Muenz DG Archer PG Jackson WC Hamstra DA Hearn JW. Changes in Prostate Orientation Due to Removal of a Foley Catheter. Med Phys (2018) 45(4):1369–78. doi: 10.1002/mp.12830 29474748

[14] Dekura Y Nishioka K Hashimoto T Miyamoto N Suzuki R Yoshimura T. The Urethral Position may Shift Due to Urethral Catheter Placement in the Treatment Planning for Prostate Radiation Therapy. Radiat Oncol (2019) 14. doi: 10.1186/s13014-019-1424-8 PMC6909476 31831045

[15] Kataria T Gupta D Goyal S Bisht SS Chaudhary R Narang K. Simple Diagrammatic Method to Delineate Male Urethra in Prostate Cancer Radiotherapy: An MRI Based Approach. Br J Radiol (2016) 89. doi: 10.1259/BJR.20160348 PMC5604912 27748126

[16] Zakian KL Wibmer A Vargas HA Alberts E Kadbi M Mychalczak B. Comparison of Motion-Insensitive T2-Weighted MRI Pulse Sequences for Visualization of the Prostatic Urethra During MR Simulation. Pract Radiat Oncol (2019) 9:e534–40. doi: 10.1016/J.PRRO.2019.06.009 PMC6832802 31252087

[17] Richardson M Skehan K Wilton L Sams J Samuels J Goodwin J. Visualising the Urethra for Prostate Radiotherapy Planning. J Med Radiat Sci (2021) 68:282–8. doi: 10.1002/JMRS.485 PMC8424315 34028976

[18] Commandeur F Simon A Mathieu R Nassef M Arango JDO Rolland Y. MRI to CT Prostate Registration for Improved Targeting in Cancer External Beam Radiotherapy. IEEE J BioMed Heal Inf (2017) 21:1015–26. doi: 10.1109/JBHI.2016.2581881 27333613

[19] Pathmanathan AU Schmidt MA Brand DH Kousi E van As NJ Tree AC. Improving Fiducial and Prostate Capsule Visualization for Radiotherapy Planning Using MRI. J Appl Clin Med Phys (2019) 20:27–36. doi: 10.1002/ACM2.12529 PMC6414142 30756456

[20] Nicosia L Sicignano G Rigo M Figlia V Cuccia F De Simone A. Daily Dosimetric Variation Between Image-Guided Volumetric Modulated Arc Radiotherapy and MR-Guided Daily Adaptive Radiotherapy for Prostate Cancer Stereotactic Body Radiotherapy. Acta Oncol (2021) 60:215–21. doi: 10.1080/0284186X.2020.1821090 32945701

[21] Tocco BR Kishan AU Ma TM Kerkmeijer LGW Tree AC. MR-Guided Radiotherapy for Prostate Cancer. Front Oncol (2020) 10:616291/BIBTEX. doi: 10.3389/FONC.2020.616291/BIBTEX 33363041 PMC7757637

[22] Pham J Savjani RR Gao Y Cao M Hu P Sheng K. Evaluation of T2-Weighted Mri for Visualization and Sparing of Urethra With Mr-Guided Radiation Therapy (Mrgrt) on-Board Mri. Cancers (Basel) (2021) 13. doi: 10.3390/CANCERS13143564 PMC8307202 34298777

[23] Ma TM Neylon J Casado M Sharma S Sheng K Low D. Dosimetric Impact of Interfraction Prostate and Seminal Vesicle Volume Changes and Rotation: A Post-Hoc Analysis of a Phase III Randomized Trial of MRI-Guided Versus CT-Guided Stereotactic Body Radiotherapy. Radiother Oncol (2022) 167:203–10. doi: 10.1016/J.RADONC.2021.12.037 34979212

